# Repurposing loratadine to reverse colistin resistance in *Klebsiella pneumoniae* through targeting lipid A modification

**DOI:** 10.1080/22221751.2026.2623697

**Published:** 2026-02-12

**Authors:** Xiaoying Wu, Zhanzhe Ge, Haojie Zhan, Mengxiang Zheng, Yiming Feng, Yajun Zhai, Li Yuan, Jianhua Liu, Yushan Pan, Gongzheng Hu, Xiaoyuan Ma, Dandan He

**Affiliations:** a College of Veterinary Medicine, Henan Agricultural University, Zhengzhou, People’s Republic of China; b Ministry of Education Key Laboratory for Animal Pathogens and Biosafety, Henan Agricultural University, Zhengzhou, People’s Republic of China; c Henan Province Key Laboratory of Animal Food Pathogens Surveillance, Henan Agricultural University, Zhengzhou, People's Republic of China

**Keywords:** *Klebsiella pneumoniae*, colistin resistance, loratadine, drug repurposing, lipid A modification

## Abstract

The emergence of multidrug-resistant *Klebsiella pneumoniae* poses a significant challenge to clinical treatment and public health. Strategies combining antibiotics with FDA-approved non-antibiotic drugs have recently attracted attention as a promising approach to overcome antibiotic resistance. In this study, we systematically evaluated the synergistic effect of the antihistamine loratadine in combination with colistin against *K. pneumoniae*. Our results demonstrate that loratadine significantly restores the bactericidal activity of colistin against colistin-resistant *K. pneumoniae* both *in vitro* and *in vivo*, without increasing toxicity, while also delaying the development of colistin resistance. Mechanistic investigations using fluorescence-based assays and proteomic analysis revealed that loratadine acts as a potent adjuvant for colistin, effectively restoring its activity against colistin-resistant *K. pneumoniae* by interfering with lipid A modification. This phenomenon is further supported by the downregulation of lipid A-modifying enzyme-related protein EptB. In addition, the combination of loratadine and colistin disrupts the double-layer membrane barrier, leading to proton motive force (PMF) dysregulation, reduced intracellular ATP levels, and impaired efflux pump activity. Collectively, this study highlights the potential of drug repurposing as an effective strategy to combat antimicrobial resistance and provides a foundation for the development of combination therapies against multidrug-resistant pathogens.

## Introduction

Antibiotic resistance has become a major global public health challenge, with carbapenem-resistant *Klebsiella pneumoniae* (CRKP) representing one of the most critical threats [1–4]. The dissemination of carbapenemase-encoding genes has led to a surge in CRKP infections, with resistance rates in China rising from 3% in 2005 to 21% in 2017, severely limiting available antimicrobial treatment options [5,6]. As a last-resort antibiotic, colistin (COL) has played a crucial role in the treatment of multidrug-resistant Gram-negative infections. However, its clinical efficacy has been increasingly compromised by extensive use and the global dissemination of the plasmid-mediated *mcr-1* gene, which has been widely reported not only in clinical isolates but also across animal and food-associated bacterial populations [7–9].

Given the limited development of new antibiotics, restoring the activity of existing drugs has emerged as an important strategy to combat antimicrobial resistance [10]. Combination therapies have attracted attention due to their synergistic effects, ability to expand clinical options, and potential to delay resistance [11,12]. Several studies have demonstrated the potential of such approaches: melatonin enhances the activity of multiple antibiotics, particularly COL, against MCR-expressing bacterial strains by enhancing bacterial outer membrane permeability, promoting oxidative damage, and inhibiting efflux pumping without increasing toxicity [13]; the combination of baicalin and EDTA shows a strong synergistic effect on COL, reversing resistance in *Salmonella* strains through accelerated tricarboxylic acid cycle activity, inhibition of bacterial antioxidant systems and lipopolysaccharide (LPS) modification, and suppression of effluent pump function [14]. Collectively, these studies highlight the promise of non-antibiotic agents as COL adjuvants. However, the number of effective COL adjuvants remains limited, and most candidates lack comprehensive mechanistic characterization or clear translational potential. Moreover, few FDA-approved drugs with established safety profiles have been systematically evaluated for this purpose, underscoring the need for further exploration.

With the increasing problem of antibiotic resistance, researchers have begun to explore alternative strategies to control antimicrobial resistance, including drug repurposing, combination therapies involving non-traditional antimicrobial agents, and mechanistic investigations targeting resistance determinants such as efflux pumps and membrane permeability [15–18]. In the initial screening of nearly 30 FDA-approved non-antibiotic drugs, including antihistamines and antitumor drugs, loratadine showed the most pronounced ability to reverse COL resistance. Therefore, subsequent investigations focused on the combination of loratadine and COL. Loratadine (LOR) is a second-generation antihistamine that is widely used to treat allergic conditions including allergic asthma, rhinitis, and chronic urticaria. As an FDA-approved over-the-counter drug, it has high oral bioavailability and a good safety profile with weak central inhibitory effects. In addition, due to its strong lipid solubility, LOR has a certain ability to permeate tissues, including lung tissue, which is highly compatible with common infection sites of CRKP, such as pneumonia [19,20].

In recent years, as antibiotic resistance has worsened, the antimicrobial potential of LOR has gradually gained attention. In certain cases, LOR can exert antimicrobial effects by inhibiting histamine release and reducing inflammation, particularly in skin inflammation and certain respiratory diseases [19,21]. Additionally, studies have shown that LOR can inhibit the virulence and biofilm formation of *Staphylococcus aureus* and reduce mortality in an *S. aureus* lung infection mouse model. When combined with vancomycin, LOR decreases pulmonary bacterial load and reduces inflammatory cytokine levels in bronchoalveolar lavage fluid [20]. Additionally, LOR, when used in combination with ofloxacin, can suppress the expression of key antibiotic resistance genes in the *bla* and *mec* operons [22]. Based on these observations, this study explores the potential of LOR as a novel antibiotic adjuvant in combination with COL against multidrug-resistant *K. pneumoniae*. To this end, we comprehensively evaluated efficacy, mechanism, and translational potential using *in vitro* assays (checkerboard synergy tests, time-kill curves, and drug resistance development assays), *in vivo* infection models, and mechanistic investigations including phenotypic assays, fluorescence-based analyzes, proteomics, and RT-qPCR validation.

## Materials and methods

### Bacteria and reagents

Nine *K. pneumoniae* strains used in this study are listed in Table 1. All strains were stored in Luria–Bertani (LB, Beijing Aoboxing Bio-technology, Beijing, China) broth supplemented with 30% glycerol at −80°C. COL (88.7% purity, purchased from Dalian Meilunbio, China) was dissolved in sterilized deionized water, and LOR (98% purity, purchased from Shanghai Aladdin, China) was dissolved in DMSO. In addition, doxycycline (97% purity) and cefothifur (98% purity) were purchased from Solarbio (Beijing, China). Ciprofloxacin (98% purity), enrofloxacin (98% purity), tigecycline (98% purity), florfenicol (98% purity), and amikacin (97% purity) were purchased from OriLeaf (Shanghai, China). Except for florfenicol was dissolved with 50% sterilized deionized water, 25% ethanol and 25% N, N-dimethylformamide, and the rest was dissolved in sterilized deionized water.

**Table 1.** Evaluation of LOR in combination with eight antibiotics, and MIC (µg/mL) and FICI of LOR and COL combined against *K. pneumoniae* strains.

Sources	IDs	Resistance mechanism	Drug	MICs (µg/mL)		FICI	Interaction
				Alone	Combination		
Environment	JX21CTR26	*oqxB*, *oqxA*, *qnrB4*	CIP/LOR	128/512	64/128	0.75	additive
		*oqxB*, *oqxA*, *qnrB4*	ENR/LOR	256/512	128/64	0.625	additive
		*tet(M)*, *tet(D)*	DOX/LOR	256/512	512/4	2.0078	antagonism
		unknown	TGC/LOR	4/512	2/256	1	additive
		*floR*	FFC/LOR	128/512	128/4	1.0078	irrelevance
		*bla*_SHV-229_, *bla*_TEM-1_, *bla*_DHA-1_	CEF/LOR	512/512	128/128	0.5	synergy
		*aadA1*, *aph(3′)-Ia*, *aac(3)-IVa*, *aph(4)-Ia*, *armA*	AMK/LOR	512/512	512/32	1.0625	irrelevance
		*mgrB*, *pmrD*, *pmrB*, and *crrB* mutation	COL/LOR	16/512	2/4	0.1328	synergy
Environment	JX21CTR30	*mgrB*, *pmrD*, *pmrB*, and *crrB* mutation	COL/LOR	16/512	1/4	0.0703	synergy
Chicken	JX21CTR16	*mcr-1*	COL/LOR	4/512	1/16	0.2813	synergy
Rabbit	KP1Q	*mcr-1*	COL/LOR	8/512	2/16	0.2813	synergy
Swine	E1	unknown	COL/LOR	8/512	1/8	0.1406	synergy
Swine	E4	unknown	COL/LOR	8/512	1/16	0.1563	synergy
Swine	KP1	*pmrD* mutation	COL/LOR	16/512	1/16	0.0938	synergy
Human	KP9	*crrA*, *crrB* mutation	COL/LOR	16/512	2/32	0.1875	synergy
Human	KH41127-6	unknown	COL/LOR	16/512	1/4	0.0703	synergy

Note: CIP: ciprofloxacin; ENR: enrofloxacin; DOX: doxycycline; TGC: tigecycline; FFC: florfenicol; CEF: cefothifur; AMK: amikacin; LOR: loratadine; COL: colistin.

### Antimicrobial susceptibility testing

The minimum inhibitory concentrations (MICs) value were determined according to the micro broth dilution method recommended by CLSI guideline [23]. COL and LOR were two-fold serial dilution with Mueller-Hinton Broth (MHB, Beijing Aoboxing Bio-technology, Beijing, China). Adjust the logarithmic growth phase bacterial suspension to 1 × 10^6^ CFU/mL and mix with the drug in a 96-well microtiter plate (Servicebio, Wuhan, China). Incubate at 37°C for 16–18 h. The MIC value of COL or LOR was determined as the lowest concentration that inhibits the growth of visible bacteria in the form of turbidity. A positive control (with bacterial suspension only) and a negative control (with MHB medium only) were set up to verify the validity of the test. All trials were repeated at least three times. *K. pneumoniae* ATCC 700603 was used as a quality control reference strain to evaluate the availability of all antibiotics in the study.

### Checkerboard assays

Referring to the checkerboard susceptibility test in [24], the suspension of bacteria was diluted according to the above method, and the checkerboard susceptibility test of various antibiotics such as COL and LOR was carried out. The test results were read after the 96-well microtiter plate was placed in a constant temperature biochemical incubator at 37°C for 16–18 h. The trial was repeated three times in parallel.

The fractional inhibitory concentration index (FICI) was calculated to assess the interaction between two antimicrobial agents. The FICI is defined by the following formula:

$$\mathrm{FICI}=\mathrm{FI}C_{A}+\mathrm{FI}C_{B}=\mathrm{MI}C_{A\;\mathrm{in}\;\mathrm{combination}}/\mathrm{MI}C_{A\;\mathrm{alone}}+\mathrm{MI}C_{B\;\mathrm{in}\;\mathrm{combination}}/\mathrm{MI}C_{B\;\mathrm{alone}}.$$

Synergy was defined as FICI ≤ 0.5. Additive was defined as 0.5 < FICI ≤ 1. Irrelevance was defined as 1 < FICI ≤ 2. Antagonism was defined as FICI > 2 [25].

### Time-kill curves

Briefly, single colonies of two COL-resistant *K. pneumoniae* strains were first picked from MacConkey agar (Beijing Aoboxing Bio-technology, Beijing, China) plates and cultured overnight in LB broth. The cultures were then inoculated into 20 mL of LB broth (200 µL) containing either no drug (control), COL (1/2 MIC, 8 µg/mL), LOR (1/16 MIC, 32 µg/mL), a combination treatment (COL plus LOR), and positive control (8 MIC, 128 µg/mL). The cultures were incubated at 37°C with shaking at 180 rpm. Aliquots were taken at 0, 2, 4, 8, 12, and 24 h, serially diluted in sterile phosphate-buffered saline (PBS) and plated onto LB agar plates to count colony-forming units (CFUs) after overnight incubation at 37°C. As previously reported, synergy was defined as a reduction ≥2 log_10_ CFU/mL with the combination treatment compared to the most active drug alone [26,27].

### Combination disc test

Bacterial cultures (*K. pneumoniae* JX21CTR26) were diluted with LB broth to OD_600_ values of 0.1 and then plated onto LB agar plates containing 0, 16, 32, or 64 µg/mL LOR. Next, 10 µg COL discs (Liofilchem, Italy) were gently placed on the agar plate. The size of the inhibition zones we observed and recorded after 18–24 h [28].

### Post-antibiotic effect assay

*K. pneumoniae* JX21CTR26 was cultured at 37°C in LB broth to the log phase. The diluted bacteria (1 × 10^8^ CFU/mL) were exposed to LOR (0, 16, 32, or 64 µg/mL), and COL (0, 4, or 8 µg/mL) alone or in combination for 2 h at 37°C. After incubation, samples were removed by diluting 1:1,000 with LB broth. Viable counts were determined immediately after dilution. The assay was performed every 1 h for a total duration of 10 h. Post-antibiotic effect (PAE) was calculated as PAE (h) = T − C, where T is the time (in hours) required for the viable count of the drug-treated bacteria to increase tenfold from the moment of dilution, and C is the time (in hours) required for the untreated bacterial cell density to increase tenfold [28].

### Resistance study

The serial passage assays were performed using the previously described method. Briefly, dilute the overnight culture of the bacteria 1:1,000-fold into the new LB medium. Always add sub-bacteriostatic concentrations of drugs to it for induction culture. One generation is trained every 12 h, and seven generations are cultivated continuously. *K. pneumoniae* JX21CTR26 at a concentration of 10^10^ CFU/mL was spread onto LB agar plates containing COL alone (0, 0.5, 1, 2, 4, 8, 16, 32, 64, 128, 256, 512 µg/mL) or in combination with various concentrations of LOR (0, 8, 16, 32, 64, 128 µg/mL). The plates were incubated at 37°C for 72 h. The mutant prevention concentration (MPC) was defined as the lowest concentration of COL that inhibited visible bacterial growth for each corresponding LOR concentration [29].

### Biofilm inhibition and clearance

Biofilm formation assay: *K. pneumoniae* strains KP1 and JX21CTR26 were cultured overnight in LB broth under a constant temperature shaker at 37°C. Transfer the culture with LB broth 1:100 to sterile 24-well plates containing silica gel sheets with 1 mL of bacterial suspension per well. Each condition is tested three times. Sterile LB broth was used as a negative control. After 48 h of incubation at 25°C, the plates were stained with 0.01% crystal violet. The OD_600_ was measured to detect the biofilm formation capacity. Biofilm-forming ability was categorized based on optical density (OD) values compared to the negative control OD cutoff (ODc): OD > 4ODc represents strong biofilm formation (+++), 2ODc < OD < 4ODc represents moderate biofilm formation (++), ODc < OD < 2ODc represents weak biofilm formation (+), OD < ODc represents no biofilm formation [30].

Inhibition: The drug combinations diluted with LB broth and bacterial solutions were mixed 1:1 by volume, placed in 24-well plates, and incubated at 25°C for 48 h, crystal violet staining, and absorbance was measured.

Clearance: After culturing mature biofilms in 24-well plates, the plankton bacteria were washed away, and each group of drugs was diluted with LB broth, added 24-well plates sequentially, and incubated at 25°C for 24 h.

Crystal violet staining, and the absorbance was determined. The biofilm inhibition or clearance rate was calculated using the following formula:

Inhibitionorclearancerate(%)=(A1−A2)/A1×100%

A1: Absorbance of the control group (bacteria with no treatment). A2: Absorbance of the treatment (bacteria plus antibiotic).

### Biochemical factors analysis

Biochemical assays were pretreated according to a similar process. Overnight cultures of *K. pneumoniae* KP1 and JX21CTR26 were washed with PBS and suspended to an OD_600_ of 0.5, incubated in groups for 1 h with drugs, followed by incubation with fluorescent probes for 30 min in the dark. Subsequently, it was transferred to a 96-well white plate. Fluorescence intensity was measured by the Infinite M200 microplate reader (Tecan).

#### Outer membrane permeability

1-N-phenylnaphthylamine (NPN) with the excitation wavelength of 350 nm and emission wavelength of 420 nm was used to evaluate the outer membrane permeability [31].

#### Cell membrane integrity

Fluorescence intensity of propidium iodide (PI)-labeled cells in the presence of increasing concentrations of drugs was measured with the excitation wavelength of 535 nm and emission wavelength of 615 nm [32].

#### Determination of proton motive force (PMF)

*K. pneumoniae* KP1 and JX21CTR26 were treated according to the above methods. The dissipated membrane potential (or intracellular pH gradient, ΔpH) of bacteria was determined using the fluorescent probe 3,3-dipropylthiadicarbocyanine iodide (DiSC_3_(5), 0.5 µM) or the pH-sensitive fluorescent probe 2′,7′-Bis (carboxyethyl)-5(6)-carboxyfluorescein acetoxymethyl ester (BCECF-AM, 20 µM) at an excitation wavelength of 622 nm (or 488 nm) and an emission wavelength of 670 nm (or 535 nm).

#### Intracellular ATP assay

Intracellular ATP levels of *K. pneumoniae* KP1 and JX21CTR26 were determined strictly according to the Enhanced ATP assay kit (Beyotime, China), and the fluorescence intensity under OD_600_ was measured with a multifunctional microplate reader to evaluate intracellular ATP levels [33].

#### Etbr activity

The fluorescent probe ethidium bromide (EtBr) was added to the prepared bacterial suspension, and the fluorescence intensity was measured under excitation and emission wavelengths of 530 and 600 nm, respectively. The activity of the intracellular efflux pump was then evaluated, described by Viveiros et al. [34].

#### ROS levels

The levels of reactive oxygen species (ROS) were measured with 2′,7′-dichlorodihydrofluorescein diacetate (DCFH-DA), following the manufacturer's instructions (Beyotime). After incubation, fluorescence intensity was measured with an excitation wavelength of 488 nm and emission wavelength of 525 nm [35]. The ROS scavenger N-acetyl cysteine (NAC) with a final concentration of 10 mM was added for checkerboard determination.

### Scanning electron microscopy (SEM)

Single colonies of *K. pneumoniae* KP1 were picked and cultured overnight in LB broth. The cultures were then inoculated into a new LB broth containing no drug (control), COL (8 µg/mL), LOR (64 µg/mL), or a combination of COL and LOR (8 + 32 or 64 µg/mL). After 4 h of incubation, the bacterial suspensions were washed twice with PBS. The cells are collected by centrifugation, fixed with 2.5% glutaraldehyde, prepared for SEM imaging. Final images were obtained by Wuhan Servicebio Co., Ltd (Wuhan, China).

### Bacterial viability assay

Overnight cultures of *K. pneumoniae* JX21CTR26 were washed and then suspended with PBS solution to an OD_600_ value of 0.5 and dosed in groups and incubated at 37°C for 4 h, followed by 30 min incubation with SYTO-9 and propidium iodide (PI) probes (Thermo Fisher Scientific) in the dark [32]. Subsequently, 10 µL of the stained suspension were placed on a slide and observed using a fluorescence microscope.

### Effect of exogenous LPS supplementation on the MIC value of COL

Cultivate JX21CTR26 to the logarithmic growth phase. The final concentration of LOR in the fixed bacterial solution was 32 µg/mL, and the checkerboard susceptibility test of LPS and COL was performed.

### Swimming motility experiment

A 0.3% semi-solid medium was prepared using agar powder and LB broth, and 2 µg/mL of COL and 16 or 32 µg/mL LOR were added to it individually or together by test grouping. 2 µL of *K. pneumoniae* JX21CTR26 suspension (OD_600_ = 0.5) was inoculated on the medium and incubated at 37°C for 48 h. The diameter of the swimming colony was measured using a vernier caliper and photographs were taken to assess the motility of the bacteria. Untreated plates were used as negative controls. The experiment was repeated three times [30].

### Proteomic analysis

To investigate the effects of COL (1/2 MIC, 8 µg/mL) and LOR (1/16 MIC, 32 µg/mL) on the expression of *K. pneumoniae*, the strain JX21CTR26 underwent proteomic analysis. The dosed cultures were washed with PBS solution, then collected, frozen in liquid nitrogen, and sent to Shanghai OE Biotech Co., Ltd. (Shanghai, China) for LC-MS/MS analysis. Database searches are used to determine the type of protein. The GO and KEGG enrichment methods were used for bioinformatics analysis of differentially expressed proteins. Data are available via ProteomeXchange with identifier PXD071946.

### RT-qPCR analysis

*K. pneumoniae* JX21CTR26 were cultured to an OD_600_ of 0.5 and incubated at 37°C for 2 h with COL (1/2 MIC, 8 µg/mL) and LOR (1/16 MIC, 32 µg/ml) alone or in combination. For specific steps, refer to previous studies [30,36]. The RT-PCR primer sequences used to detect the expression level of gene mRNA are shown in Table S1.

### Animal study

For the *in vivo* experiments, both acute toxicity testing in mice and evaluation of the therapeutic efficacy of LOR and COL against *K. pneumoniae* JX21CTR26 infection were conducted. Four-week-old KM male and female mice suitable for toxicology testing with individual variations (approximately 20 g), and six-week-old BALB/c female mice with good repeatability and low error were purchased were purchased from the Huaxing Experimental Animal Center (Zhengzhou, China). Mice were acclimated to the controlled environment for one week prior to the experiments.

#### Haemolytic activity

Mouse red blood cells (RBC) were washed three times with normal saline. 500 µL of a 2% red blood cell suspension was mixed with 500 µL of LOR at various concentrations (8–128 µg/mL) and its combination with 8 µg/mL COL in 1.5 mL eppendorf (EP) tubes. Ultrapure water and normal saline were used as positive control (PC) and negative control (NC), respectively. 10% DMSO was used as a solvent control. After incubation at 37°C for 2 h, the samples were centrifuged at 10,000 × g for 5 min. A 200 µL aliquot of the supernatant was transferred to a 96-well plate, and the absorbanceat at 540 nm was measured using a Multiskan GO Microplate Reader (Thermo Fisher Scientific). The haemolysis rate was calculated according to the following formula:

$$\mathrm{Haemolysis}(\text{\%})=[(OD_{\mathrm{Sample}}-OD_{\mathrm{NC}})/(OD_{\mathrm{PC}}-OD_{\mathrm{NC}})]\times 100\text{\%}$$

#### In vivo toxicity evaluation

To evaluate *in vivo* toxicity, four-week-old KM female mice (*n* = 5) and KM male mice (*n* = 5) were each administered LOR at a dose of 50 mg/kg. The general condition and body weight of the mice were monitored daily for 14 days.

#### Mouse infection model

A mouse model of endonasal pulmonary infection was used to determine the synergistic effect of LOR in combination with COL *in vivo*. Six-week-old BALB/c female mice (*n* = 7 per group) were positioned upright with their heads tilted back, and 50 µL of JX21CTR26 suspension (approximate 10^8^ CFUs) was instilled into the nasal cavity. After 1 h post-infection, mice received intraperitoneal injections every 12 h with either PBS (control), COL (5 mg/kg), LOR (2.5 mg/kg), or a combination treatment (5 mg/kg of COL + 2.5 mg/kg of LOR). At 60 h post-infection, the mice were blood-sampled and then sacrificed. Serum is isolated from the collected blood for later use. Six mice were selected for lung tissue homogenization in each group. Diluted tissue samples were plated on McConkey agar to determine bacterial counts. Additionally, one of each group were selected for histopathological analysis. Lung tissue was fixed with 4% paraformaldehyde and sent to Wuhan Servicebio Co., Ltd. for histological examination. Three samples were randomly selected from the isolated serum in each group, and the levels of inflammatory cytokines, including tumor necrosis factor-α (TNF-α), interleukin-1β (IL-1β) and interleukin-10 (IL-10) in lung tissues were determined using ELISA kits (BioLegend, USA) according to the manufacturer's instructions [37].

### Statistical analysis

Employing GraphPad Prism software (version 10.0), we performed all statistical analyzes. Inter-group differences were assessed by an unpaired t-test (for two groups) or one-way ANOVA (for more than two groups). Statistical significance was determined as follows: *p < 0.05, **p < 0.01, ***p < 0.001, and ****p < 0.0001. Unless otherwise noted, all data in this article are represented as the mean ± standard deviation (SD) of at least three separate experiments.

## Results

### Synergistic activity of LOR with COL

**Figure 1.**
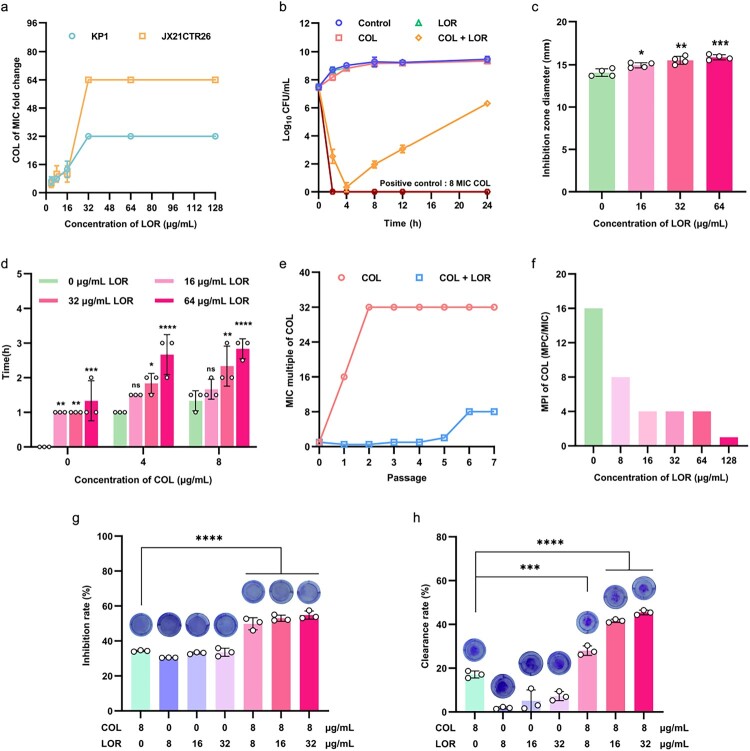
Synergistic effects of LOR in combination with COL on *K. pneumoniae in vitro*. (a) Dose-effect relationship between LOR and COL. (b) Time-kill curves of *K. pneumoniae* JX21CTR26. The control group was treated without drug. In the COL group, 1/2 MIC (8 µg/mL) COL was added alone. In the LOR group, 1/16 MIC (32 µg/mL) LOR was added alone. In the COL + LOR group, a combination of 1/2 MIC COL and 1/16 MIC LOR was used. (c) Effects of different concentrations of LOR in combination with COL on the antibacterial activity of *K. pneumoniae* JX21CTR26. (d) Post-antibiotic effect of COL, alone and in combination with various concentrations of LOR on *K. pneumoniae* strain. (e) Evaluation of the development of resistance to COL alone or in combination with LOR for seven consecutive generations at subinhibitory concentrations. (f) Mutation prevention index (MPI) of *K. pneumoniae* JX21CTR26 to COL in the presence of different concentrations of LOR. (g,h) Efficiency of biofilm inhibition and clearance among different treatment groups. Data represent mean ± SD of at least three biological replicates and statistical differences were analyzed by non-parametric one-way ANOVA (*p < 0.05, **p < 0.01, ***p < 0.001, ****p < 0.0001).

To evaluate the potential efficacy of LOR, a checkerboard test was performed using LOR with eight antibiotics against multidrug-resistant bacteria JX21CTR26 including COL, ciprofloxacin, enrofloxacin, doxycycline, tigecycline, florfenicol, ceftiofur, and amikacin. It was found that LOR only potentiated the activity of COL (Table 1, Figure S1). Next, we randomly selected nine strains of *K. pneumoniae* with different resistance mechanisms isolated from different sources to verify the effect of the combination of LOR and COL. The checkerboard assay demonstrated FICI < 0.5 for all tested strains when combining COL with LOR (Table 1). This confirms the antibacterial synergy of combination therapy. The dose-response relationship between LOR and COL showed that with the increase of LOR concentration, the fold change of the MIC of COL rose and eventually plateaued. At LOR concentrations of 32 µg/mL (1/16 MIC), the MIC reduction of COL is maximized. This provides a basis for the selection of LOR concentrations in subsequent experiments (Figure 1(a)). Time-kill curves showed that neither LOR nor COL alone killed the bacteria, whereas the combination of 32 µg/mL LOR and 8 µg/mL COL rapidly reduced the number of bacteria by more than 2 log_10_ CFU/mL (Figure 1(b) and S2). And after 24 h, the colony count in the combined group was still >2 log_10_ CFU/mL compared to the COL monotherapy at this time. This indicates that the addition of LOR can restore the bactericidal effect of COL.

The combined disc test also demonstrated the synergistic potential between LOR and COL, as evidenced by a significantly larger zone of inhibition (Figure 1(c) and S3). Moreover, significant post-antibiotic effect (PAE) enhancement observed in combination therapy (Figure 1(d)), reflected in the extended time for bacterial growth to reach the logarithmic phase after drug removal (Figure S4). This suggests that LOR, when combined with COL, prolongs the effect on bacterial growth.

### LOR prevents the evolution of COL resistance

Preventing the evolution of bacterial resistance is crucial. Resistance levels to COL increased significantly when exposed to subinhibitory concentrations, and its MIC value increased 32 folds over seven consecutive passages (Figure 1(e)). Importantly, when LOR and COL were combined, the level of resistance increased only slightly by a fold of 8. Mutant prophylaxis concentration (MPC) serves as an key indicator of resistance changes [38]. LOR reduced the mutation prevention index (MPI) of COL by 16 folds, i.e. from 16 to 1 in a dose-dependent manner (Figure 1(f)), suggesting that the combination of LOR and COL is effective in delaying the onset of COL resistance in *K. pneumoniae*.

### LOR enhances antibiofilm activity when combined with COL

The formation of biofilms significantly enhances bacterial resistance to antibiotics and increases their virulence and pathogenicity, posing a major challenge in the treatment of clinical infections [39]. In this study, the biofilm-formation abilities of *K. pneumoniae* strains KP1 and JX21CTR26 were assessed, with KP1 showing moderate biofilm formation and JX21CTR26 exhibiting strong biofilm formation (Table S2). The antibiofilm activities of LOR, COL and their combinations were evaluated using crystal violet staining. When administered alone at a concentration of 8 µg/mL, COL exhibited a biofilm formation inhibition rate of 34.30% and a clearance rate of 18.57%, while LOR alone showed inhibition and clearance rates of no more than 33.57% and 7.90%, respectively. Notably, the combination of COL (8 µg/mL) and LOR (32 µg/mL) significantly inhibited biofilm formation (49.88%) and enhanced clearance (45.44%) (Figure 1(g,h)). These findings indicate that the COL-LOR combination exerts a synergistic effect, significantly inhibiting biofilm formation and enhancing clearance compared to either agent alone.

### Mechanism of LOR in restoring susceptibility to COL-resistant K. pneumoniae

**Figure 2.**
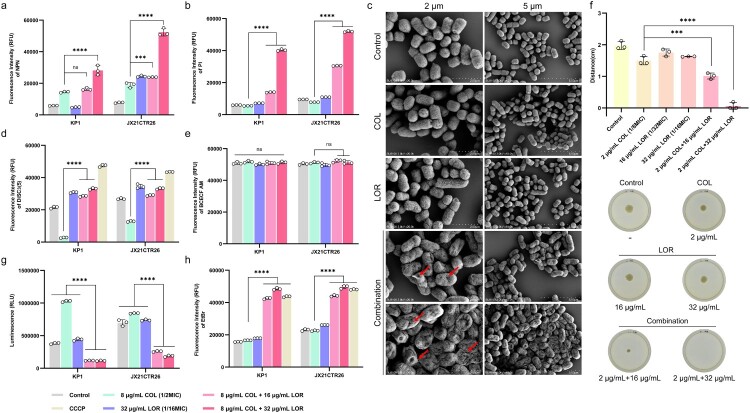
LOR and COL affected membrane integrity, proton motive force (PMF), intracellular ATP levels and efflux pump activity. *K. pneumoniae* strains KP1 and JX21CTR26 were treated with COL and LOR alone or in combinations and the properties involved were quantified as follows: outer membrane permeability via NPN uptake (a); inner membrane integrity via PI influx (b); membrane potential (Δ*φ*) using DiSC_3_(5) fluorescence (d); proton gradient (ΔpH) with BCECF-AM (e); intracellular ATP levels based on the luminescence signals (g); and efflux pump activity by EtBr accumulation (h). Morphological changes of the bacterial cell membrane were elucidated by transmission electron microscopy following different treatment group (scale bar: 2 and 5 µm) (c). The changes in PMF were indirectly evaluated by measuring the motility of bacteria (f). Data represent mean ± SD of three biological replicates and statistical differences were analyzed by non-parametric one-way ANOVA (*p < 0.05, **p < 0.01, ***p < 0.001, ****p < 0.0001).

Following confirmation of LOR restored COL bactericidal activity against COL-resistant *K. pneumoniae*, we investigated the underlying mechanisms. To evaluate bacterial membrane effects, fluorescent probes NPN (outer membrane permeability) and PI (inner membrane integrity) were utilized. Compared to COL treatment alone, the combination of COL and LOR (especially 8 µg/mL COL + 32 µg/mL LOR) markedly enhanced the fluorescence intensities of both NPN (KP1 increased by 1.94 folds, JX21CTR26 increased by 2.77 folds, *p* < 0.0001) and PI (KP1 increased by 7.30 folds, JX21CTR26 increased by 6.65 folds, *p* < 0.0001), suggesting LOR's inclusion disrupts both membrane barriers (Figure 2(a,b)). SEM analysis provided further evidence supporting these observations (Figure 2(c)). Untreated KP1 bacteria displayed typical rod-shaped morphology. COL or LOR alone only caused slight surface wrinkling in some bacterial cells. In contrast, the combination treatment induced severe cellular shrinkage and collapse, indicative of extensive membrane damage and bacterial death. To further validate the disruption of membrane integrity by COL, LOR, and their combinations, bacterial live/death staining was performed using the fluorescent dyes SYTO-9 and PI (Figure S5). COL (8 µg/mL) or LOR (32 µg/mL) alone had little effect on bacterial survival compared to controls. In contrast, the combination of COL and LOR significantly increased the number of dead bacteria stained red by the PI probe. These results suggest that the combination of LOR and COL has a strong membrane-disrupting effect. Given the specific binding mechanism between COL and LPS, we further evaluated the impact of exogenous LPS supplementation on the antibacterial activity of COL alone and in combination with LOR (Table S3). The results showed that with the increase of LPS concentration, the minimum inhibitory concentration (MIC) value of the two treatment groups showed an upward trend. When the LPS concentration reached 512 µg/mL, the MIC of the COL monotherapy group increased by 8-fold, while the MIC of the LOR–COL combination increased by 16-fold. These results indicate that exogenous LPS competitively attenuates the antibacterial activity of COL, and the more pronounced MIC increase observed for the combination treatment suggests that LOR enhances the interaction between COL and LPS, supporting the involvement of LPS (lipid A) in the synergistic mechanism.

Generally, disruption of bacterial membranes often disturbs the proton motive force (PMF), which is essential for maintaining intracellular homeostasis. This occurs due to increased membrane permeability and imbalance in transmembrane exchange. To explore this, membrane potential (Δ*φ*) and pH gradient (ΔpH) were measured using the fluorescent probes DiSC_3_(5) and BCECF-AM. The combination of LOR and COL significantly increased the fluorescence intensity of the membrane potential, indicating membrane depolarization (Figure 2(d) and S6, KP1 increased by 12.20 folds, JX21CTR26 increased by 2.58 folds, *p* < 0.0001). The ΔpH remained largely unchanged, indicating that the combination of LOR and COL did not damage the ΔpH of the cell membrane (Figure 2(e), COL group vs combination (8 + 32 µg/mL) group – mean fluorescence intensity KP1: 51762 ± 675.3 vs 51285 ± 791.6, *p* = 0.9537; JX21CTR26: 51226 ± 542.8 vs 51298 ± 1063, *p* > 0.9999). These results suggest that LOR in combination with COL disrupts PMF homeostasis primarily through Δ*φ* depolarization. Additionally, bacterial swimming motility was evaluated in soft agar assays as an indirect phenotypic correlate potentially associated with changes in PMF. The results revealed that COL-LOR combination significantly impaired bacterial motility compared to COL alone (Figure 2(f)). Since PMF disruption can impair ATP synthesis and cellular energy metabolism, intracellular ATP levels were also measured. The results showed that COL-LOR combination markedly depleted intracellular ATP levels compared to COL alone. (Figure 2(g)). This reduction in ATP levels prompted further investigation into energy-dependent processes, such as efflux pump activity. Fluorescence assays indicated that efflux activity was significantly inhibited in the combination group compared to the single-drug groups, leading to increased intracellular accumulation of the drug (Figure 2(h)). Reactive oxygen species (ROS) levels were also measured to assess oxidative stress. However, no significant increase in ROS levels was observed following the combination treatment, indicating that oxidative damage is not a major contributor to the enhanced antibacterial activity (Figure S7).

### Mechanism of synergy between LOR and COL

**Figure 3.**
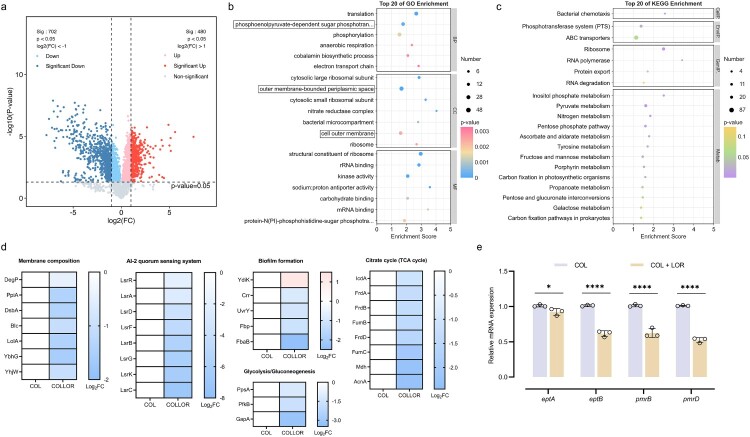
The mechanism of LOR reversing COL resistance was studied in *K. pneumoniae* JX21CTR26. (a) Volcano map of differentially expressed proteins (DEPs). (b) GO functional enrichment analysis of DEPs. (c) KEGG pathway enrichment analysis of DEPs. (d) Pathway impact analysis: The combination therapy affected membrane-related processes (membrane composition, biofilm formation), signaling pathways (AI-2 quorum sensing), and key metabolic processes (tricarboxylic acid cycling, glycolysis). (e) The effects of LOR and COL on the expression of genes (*eptA*, *eptB*, *pmrB*, *pmrD*) in *K. pneumoniae* were verified by RT-qPCR.

To elucidate the molecular mechanism by which LOR restores COL sensitivity, we performed a proteomic analysis of *K. pneumoniae* JX21CTR26 after 2 h of treatment with COL alone (8 µg/mL) or in combination with LOR (32 µg/mL). Proteomic analysis identified 480 upregulated differential proteins and 702 downregulated DEPs (>2.0 folds) (Figure 3(a)). GO/KEGG enrichment showed that DEP are involved in ribosomal function, bacterial microcompartments, inner and outer membrane associations, tricarboxylic acid cycle (TCA cycle), glycolysis, and quorum sensing system (Figure 3(b,c)). Specifically, a variety of proteins related to bacterial membrane composition showed significant changes, such as Blc, LolA, YbhG. The abundance of these proteins experienced a 2.66–3.05 folds decline after LOR stimulation (Figure 3(d)). Notably, the expression level of phosphorylethanolamine (pEtN) transferase YhjW, which modifies lipid A, was also down-regulated by 2.03 folds. The expression of proteins (including PpsA, PfkB, GapA) of related enzymes in the glycolytic pathway, as well as proteins related to the tricarboxylic acid cycle pathway, such as IcdA, FrdABD, FumBC, Mdh, and AcnA, were down-regulated. The overall expression levels of proteins related to biofilm formation, such as YdiK (up-regulation), FbaB, UvrY, Fbp, Crr, and the core regulation and transporter (LsrABCDFGKR) of the Autoinducer 2 (AI-2) quorum sensing system, decreased significantly.

To further verify the mechanism indicated by proteomics, RT-qPCR was performed to measure the expression levels of relevant genes (Figure 3(e)). Compared with the COL monotherapy group, the combination of LOR and COL significantly down-regulated the expression of lipid A modification-related gene *eptA* by 1.10 folds and *eptB* by 1.65 folds. This is consistent with the results observed in proteomics as well as with fluorescent probes on the inner and outer membranes. At the same time, the combined use altered the expression of genes related to the mechanism of COL resistance (*pmrB* down-regulated 1.62 folds, and *pmrD* down-regulated 1.94 folds).

Under the stress test of exogenous addition of high concentrations (10 mM) of Ca^2+^/Mg^2+^, COL and LOR still had a synergistic effect (Table S4). This suggests that LOR's synergistic effect on COL does not depend on a low-cationic environment.

### Safety evaluation of LOR

**Figure 4.**
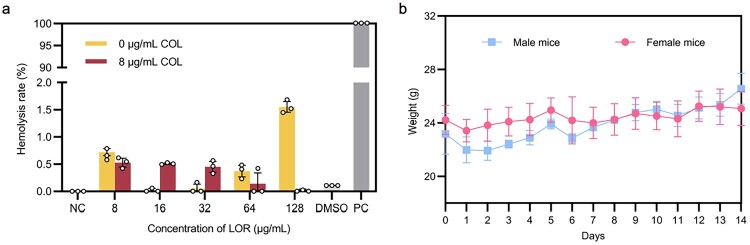
Hemocompatibility and acute toxicity assessment of LOR. (a) Mouse RBC hemolysis by LOR alone or combined with COL (8 µg/mL). (b) Acute toxicity in mice: Body weight changes 14 days post high-dose LOR (50 mg/kg) injection. Data represent mean ± SD of three biological replicates.

To evaluate the safety of LOR combined with COL, mouse red blood cells (RBC) hemolysis was assessed under the condition of co-administration of 8 µg/mL COL and different concentrations of LOR (Figure 4(a)). The results showed that LOR combination with COL maintained low RBC hemolysis (≤3%), demonstrating favorable blood compatibility. In addition, acute toxicity assessment was conducted in a mouse model. Following intraperitoneal injection of LOR at 50 mg/kg, no significant changes in body weight were observed over a 14-day period, and no signs of toxicity or adverse effects were detected (Figure 4(b)). These results suggest that LOR, either alone or in combination with COL, is safe at test concentrations.

### LOR enhances the therapeutic effect of COL in an in vivo infection model

**Figure 5.**
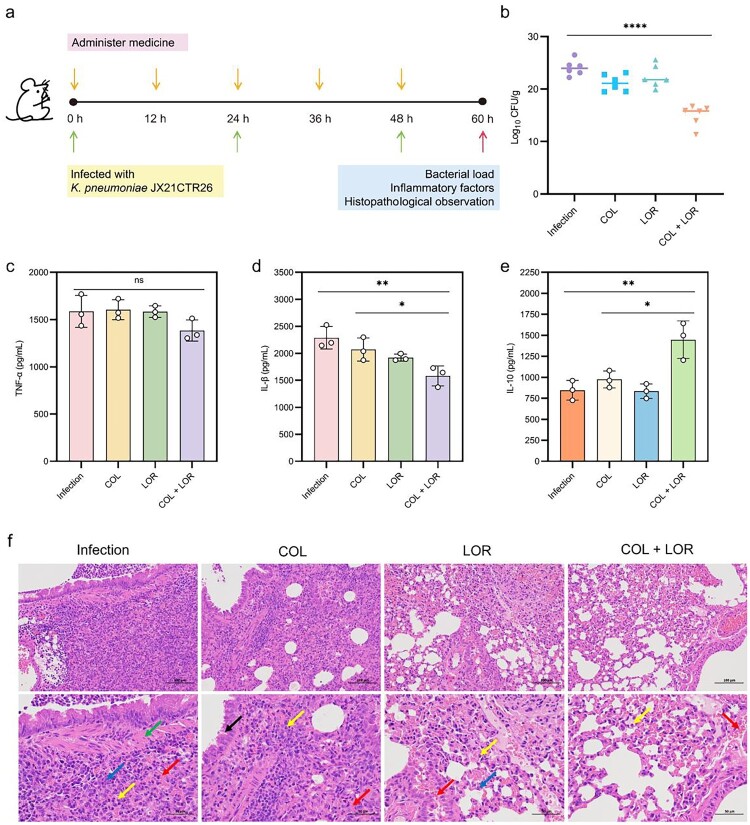
LOR-COL synergy in *K. pneumoniae* pneumonia mouse model. (a) Schematic of mouse intranasal pneumonia model. Intranasal infection with *K. pneumoniae* JX21CTR26 occurred once every 24 h (green arrow). Treatment was administered intraperitoneally every 12 h (yellow arrow). The various indicators of the mice were detected after 60 h (red arrow). (b) Lung bacterial burden (CFU) in *K. pneumoniae* JX21CTR26-infected mice after monotherapy and combination therapy. (c–e) Serum inflammatory cytokines (TNF-α, IL-1β, IL-10) quantified by ELISA: control, COL (5 mg/kg), LOR (2.5 mg/kg), and combination therapy (5 + 2.5 mg/kg). (f) Lung histopathology (H&E staining): neutrophil infiltration (yellow arrow), hemorrhage (red arrow), thickening of the alveolar wall (green arrow), a small number of cell debris scattered (blue arrow), and a small number of bronchial epithelial cells are irregularly arranged (black arrow). Data represent mean ± SD of three biological replicates and statistical differences were analyzed by non-parametric one-way ANOVA (*p < 0.05, **p < 0.01, ***p < 0.001, ****p < 0.0001).

Given that LOR can significantly restore the bactericidal effect of COL against *K. pneumoniae in vitro*, their therapeutic effect *in vivo* was investigated using a mouse model of pneumonia infection (Figure 5(a)). Notably, monotherapy with either COL or LOR failed to effectively control the infection. In contrast, combination therapy significantly reduced bacterial load in the lungs, indicating a clear therapeutic benefit (Figure 5(b) and Table S5). To evaluate the immunomodulatory effects of LOR combined with COL, we evaluated the levels of pro-inflammatory factors TNF-α, IL-1β, and anti-inflammatory factor IL-10 in serum from mice (Figure 5(c–e)). The results showed that the combination of 5 mg/kg COL + 2.5 mg/kg LOR reduced the level of pro-inflammatory factors by 1.16 and 1.31 folds, respectively, compared with the COL monotherapy group, and increased the level of the anti-inflammatory factor IL-10 (compared with the COL monotherapy group, the combination group increased by 1.49 folds), indicating that the combination of LOR and COL was involved in the regulation of the inflammatory response during treatment.

In parallel, lung histopathological analysis further confirmed the treatment effect. Compared to COL or LOR alone, combination therapy significantly reduced neutrophil infiltration and thickening of the alveolar wall (Figure 5(f), Table S6). Overall, these results indicate that LOR not only enhances the antibacterial efficacy of COL but also helps attenuate the associated inflammatory response, thereby improving therapeutic outcomes against *K. pneumoniae* infection *in vivo*.

## Discussion

COL resistance represents a complex and escalating global health concern, particularly in the context of multidrug-resistant (MDR) bacterial infections, where COL is often regarded as the last line of defense [40,41]. In response to the limited development of new antibiotics, repurposing FDA-approved non-antibiotic drugs as antibiotic adjuvants has emerged as a promising strategy to restore antimicrobial efficacy and mitigate resistance development [42–44]. In this study, LOR, a clinically approved second-generation antihistamine, was identified through preliminary screening as a potent adjuvant capable of restoring COL activity against resistant *K. pneumoniae*, and its underlying mechanisms were systematically investigated.

Phenotypic assays confirmed that LOR alone does not exhibit intrinsic antibacterial activity either *in vitro* or *in vivo*. However, when combined with COL, LOR significantly enhanced the antibacterial efficacy of COL against COL-resistant *K. pneumoniae*, restored bactericidal activity, delayed resistance development, and markedly inhibited both biofilm formation and established biofilms. These findings highlight LOR as a functional antibiotic adjuvant rather than an independent antimicrobial agent.

Mechanistically, our data indicate that the synergy between LOR and COL is primarily mediated through enhanced disruption of bacterial membrane integrity. COL has been reported to exert antibacterial activity by binding to LPS and displacing divalent cations such as Mg^2+^ and Ca^2+^, leading to destabilization of the outer membrane and subsequent inner membrane damage. However, in our study, the addition of Ca^2+^ or Mg^2+^ did not affect the MIC of COL alone or the MIC of the COL + LOR combination, suggesting that the enhanced activity of the combination is not solely dependent on the low-cation environment but likely involves other mechanisms. Proteomic analysis revealed marked downregulation of outer membrane-associated lipoproteins Blc and LolA, which are essential for outer membrane biogenesis and maintenance [45,46], as well as the inner membrane protein YbhG [47], collectively indicating compromised envelope integrity that may contribute to the potentiation of COL activity by LOR. Notably, COL resistance in Gram-negative bacteria is closely associated with lipid A modification through the addition of phosphoethanolamine (pEtN) and/or 4-amino-4-deoxy-L-arabinose, which reduces the net negative charge of LPS and impairs COL binding [48,49]. In our proteomic dataset, the lipid A-modifying enzyme EptB (formerly YhjW) was significantly downregulated following LOR–COL combination treatment, suggesting that LOR may restore COL susceptibility by suppressing lipid A modification and thereby facilitating COL–LPS interaction. Additionally, proteins involved in outer membrane stress response and protein folding, including DegP, DsbA, and PpiA, whose roles have been described in previous studies [50–52], were also downregulated in our dataset. This reduction likely further destabilizes the bacterial envelope and synergistically enhances COL activity. Importantly, physiological concentrations of Ca^2+^ and Mg^2+^ are known to attenuate COL activity by competing for LPS binding sites. Our results demonstrate that the LOR–COL combination retained synergistic activity even in the presence of elevated divalent cation concentrations, indicating that the observed synergy is not dependent on artificially low cationic conditions and may remain effective under clinically relevant environments. This property enhances the translational potential of the combination therapy.

Beyond membrane disruption, the LOR–COL combination profoundly affected bacterial energy metabolism. In our proteomic analysis, key enzymes in the glycolytic pathway (PpsA, PfkB, and GapA) were significantly downregulated, consistent with their established roles in glycolysis [53–55]. Similarly, several TCA cycle-related proteins, including isocitrate dehydrogenase IcdA, fumarate reductase FrdABD, fumarate hydratase FumBC, malate dehydrogenase Mdh, and aconitase AcnA, were also downregulated, in line with their known functions in the TCA cycle [45,56–59]. These proteomic changes corresponded with a marked reduction in intracellular ATP levels, suggesting that membrane damage and metabolic disruption are mechanistically linked, resulting in impaired proton motive force (PMF) homeostasis and secondary inhibition of energy-dependent processes, including efflux pump activity.

Proteins associated with biofilm formation and regulation were also substantially altered. Specifically, YdiK was upregulated, whereas FbaB, UvrY, Fbp, and Crr were downregulated, consistent with their reported roles in biofilm formation [60–62]. Notably, the AI-2 quorum sensing system plays a central role in *K. pneumoniae* biofilm development, and in our dataset, the core regulators and transporters of this system (LsrR, LsrA, LsrD, LsrF, LsrB, LsrG, LsrK, and LsrC) were broadly downregulated [63]. These proteomic findings align closely with the *in vitro* phenotype, in which LOR combined with COL significantly inhibited and eradicated biofilms, supporting the notion that interference with quorum sensing signaling and extracellular matrix synthesis contributes to the combination's antibiofilm effect.

*In vivo*, the LOR–COL combination reduced bacterial burden and was associated with modest but consistent decreases in pro-inflammatory cytokines. These changes likely reflect attenuation of infection-driven inflammation secondary to improved bacterial clearance rather than direct immunosuppressive effects. Although the magnitude of cytokine modulation was limited, the overall trend supports a beneficial therapeutic effect by alleviating infection-associated inflammatory responses.

**Figure 6.**
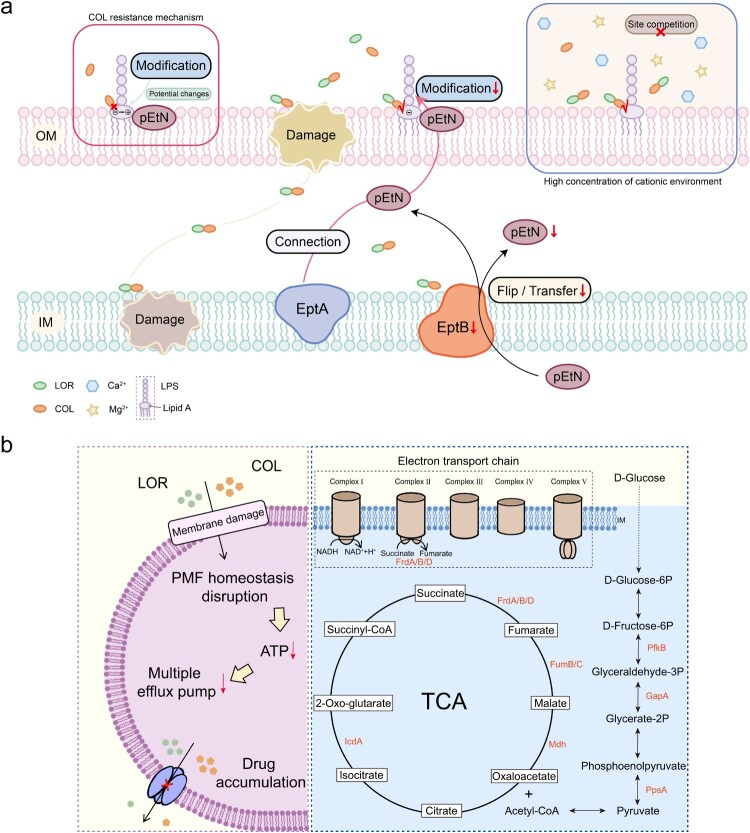
The mechanism of reversal of LOR reversal of COL-resistant *K. pneumoniae* to COL and the antibacterial mechanism of LOR combined with COL. (a) The addition of LOR inhibits the lipid A modification pathway, allowing COL to bind to LPS to exert bactericidal effects, thereby reversing the resistance of COL-resistant *K. pneumoniae*. In addition, LOR combined with COL has a competitive advantage in high cationic concentrations. (b) The combination of LOR and COL disrupts the integrity of the cell membrane, affects proton motive force, disrupts central carbon metabolism (including the tricarboxylic acid cycle, glycolysis and the electron transport chain), resulting in a decrease in ATP content, and increases the accumulation of antibiotics within the cell. Thereby synergistic enhances the bactericidal activity of COL.

Collectively, these findings suggest that LOR primarily restores COL activity against resistant *K. pneumoniae* by inhibiting lipid A modification and destabilizing bacterial membrane integrity, with downstream effects on energy metabolism, efflux pump function, and biofilm formation (Figure 6). These mechanistic insights provide a strong rationale for developing novel COL-based combination therapies and support broader drug repurposing strategies to combat COL-resistant pathogens.

## Conclusion

Through systematic evaluation of the synergistic activity of LOR and COL both *in vitro* and *in vivo*, combined with proteomic analyzes, this study demonstrates that LOR effectively reverses COL resistance in *K. pneumoniae*, primarily by inhibiting the lipid A modification pathway. As a promising non-antibiotic adjuvant, LOR offers a potential strategy to restore COL efficacy against multidrug-resistant *K. pneumoniae*. Nevertheless, several key aspects remain to be addressed. In particular, comprehensive pharmacokinetic studies, including absorption, distribution, metabolism, and excretion, are needed to better characterize the interaction between LOR and COL and to define optimal dosing regimens. Furthermore, exploration of appropriate drug delivery strategies will be essential to maximize therapeutic efficacy and support future translational applications.

## Author contributions

**Xiaoying Wu, Zhanzhe Ge**: Experimentation, Data curation, Writing-original draft, Methodology. **Xiaoying Wu, Haojie Zhan**: Software, Formal analysis, Validation. **Mengxiang Zheng, Yiming Feng**: Investigation. **Yajun Zhai, Li Yuan, Jianhua Liu, Yushan Pan, Gongzheng Hu**: Supervision, Resources. **Xiaoyuan Ma, Dandan He**: Conceptualization, Methodology, Writing-Review and Editing, Funding acquisition.

## Supplementary Material

Supplemental Material
