# Supplementary material for: Repurposing loratadine to reverse colistin resistance in Klebsiella pneumoniae through targeting lipid A modification
Source: Emerg Microbes Infect. 2026 Feb 12;15(1):2623697. doi: 10.1080/22221751.2026.2623697 (PMC12903943; doi:10.1080/22221751.2026.2623697)
Supplement: Supplemental Material [file TEMI_A_2623697_SM6730.docx]

Table S1. Primers for quantitative RT-qPCR used in this study.

| Primer | Primer sequence (5’→3’) |
| --- | --- |
| *16S rRNA-*F | GATTTAGACGCCGATGGTCTG |
| *16S rRNA-*R | CTTTGGTCACCACCGCATT |
| *eptA-*F | CCTGTTCATTCTGCTCAGCG |
| *eptA-*R | ATTTCGCCGGAGAGTAGAGC |
| *eptB-*F | GTGGTGATTGGCTACGGGAT |
| *eptB-*R | GCTCCAGATGAACAGCAACG |
| *pmrB-*F | CACGACTGCAAAGCGAACT |
| *pmrB-*R | AGGCGGGAAACCAACTGAT |
| *pmrD-*F | CTATAATGAGTGGCGTTGCGG |
| *pmrD-*R | TCGCAGAGATTGAAGCCTGT |

Table S2. Biofilm formation capacity of *K. pneumoniae* strains KP1 and JX21CTR26.

| Strain IDs | Judgment | Film-formation capacity |
| --- | --- | --- |
| KP1 | 2ODc < OD < 4ODc | moderate |
| JX21CTR26 | 4ODc < OD | strong |

Note: Biofilm formation was categorized based on OD values relative to the cut-off value (ODc) determined from the negative control. OD > 4ODc: strong (+++); 2ODc < OD < 4ODc: moderate (++); ODc < OD < 2ODc: weak (+); OD < ODc: none (−).

Table S3. Changes in MIC value after exogenous LPS supplementation.

| Strain | Drugs | COL MICs (μg/mL) | | | | | |
| --- | --- | --- | --- | --- | --- | --- | --- |
|  |  | +0LPS | +4LPS | +32LPS | +128LPS | +256LPS | +512LPS |
| JX21CTR26 | COL | 16 | 32 | 32 | 32 | 64 | 128 |
|  | COL+LOR | 2 | 4 | 4 | 8 | 16 | 32 |

Note: LOR is added at a dosage of 32 μg/mL.

Table S4. Exogenous addition of Ca^2+^ and Mg^2+^ to assess the effect on the synergistic effect of LOR and COL.

| Strain |  | Drug | MICs (μg/mL) | | FICI | Interaction |
| --- | --- | --- | --- | --- | --- | --- |
|  |  |  | Alone | Combination |  |  |
| JX21CTR26 | control | COL/LOR | 16/512 | 2/4 | 0.1328 | synergy |
|  | + Ca^2+^ | COL/LOR | 16/512 | 2/4 | 0.1328 | synergy |
|  | + Mg^2+^ | COL/LOR | 8/512 | 2/4 | 0.2578 | synergy |

Table S5. Bacterial counts after infection in the animal model.

| Groups | Samples (Bacterial counts/Lung tissue weight, Log_10_ CFU/g) | | | | | |
| --- | --- | --- | --- | --- | --- | --- |
|  | 1 | 2 | 3 | 4 | 5 | 6 |
| Infection | 4.0000/0.17 | 4.0000/0.18 | 4.3980/0.18 | 3.6990/0.16 | 4.1761/0.17 | 3.9777/0.15 |
| COL | 3.6990/0.17 | 3.9031/0.20 | 3.6990/0.19 | 4.0969/0.18 | 3.6990/0.16 | 3.8751/0.19 |
| LOR | 3.6990/0.17 | 3.9294/0.18 | 4.6021/0.18 | 3.6532/0.15 | 3.9777/0.19 | 3.9777/0.20 |
| COL + LOR | 3.1761/0.19 | 3.0000/0.19 | 3.0969/0.19 | 1.6990/0.15 | 2.6532/0.19 | 2.5441/0.16 |

Table S6. Pathological score of mouse lung sections.

| Groups | Lesions | | | | | Total score |
| --- | --- | --- | --- | --- | --- | --- |
|  | Inflammatory cell infiltration | Thickening of the alveolar walls | Hemorrhage | Extravasated blood | Necrosis |  |
| Infection | 3 | 4 | 1 | 0 | 2 | 10 |
| COL | 3 | 2 | 1 | 0 | 0 | 6 |
| LOR | 2 | 3 | 1 | 0 | 1 | 7 |
| COL + LOR | 1 | 1 | 2 | 1 | 0 | 5 |

Note: The evaluation criteria adopt a four-level grading system. Grade 0 is within the normal range, indicating that the tissue is considered normal under the study condition, taking into account factors such as the animal’s age, sex, and strain. Changes under other conditions may be considered abnormal; Grade 1 is very mild, indicating that the changes are just above the normal range; Grade 2 is mild, indicating that lesions can be observed but are not severe; Grade 3 is moderate, indicating that the lesion is obvious and likely more severe; Grade 4 is severe, indicating that the lesion is very severe (the lesion has occupied the entire tissue and organ).


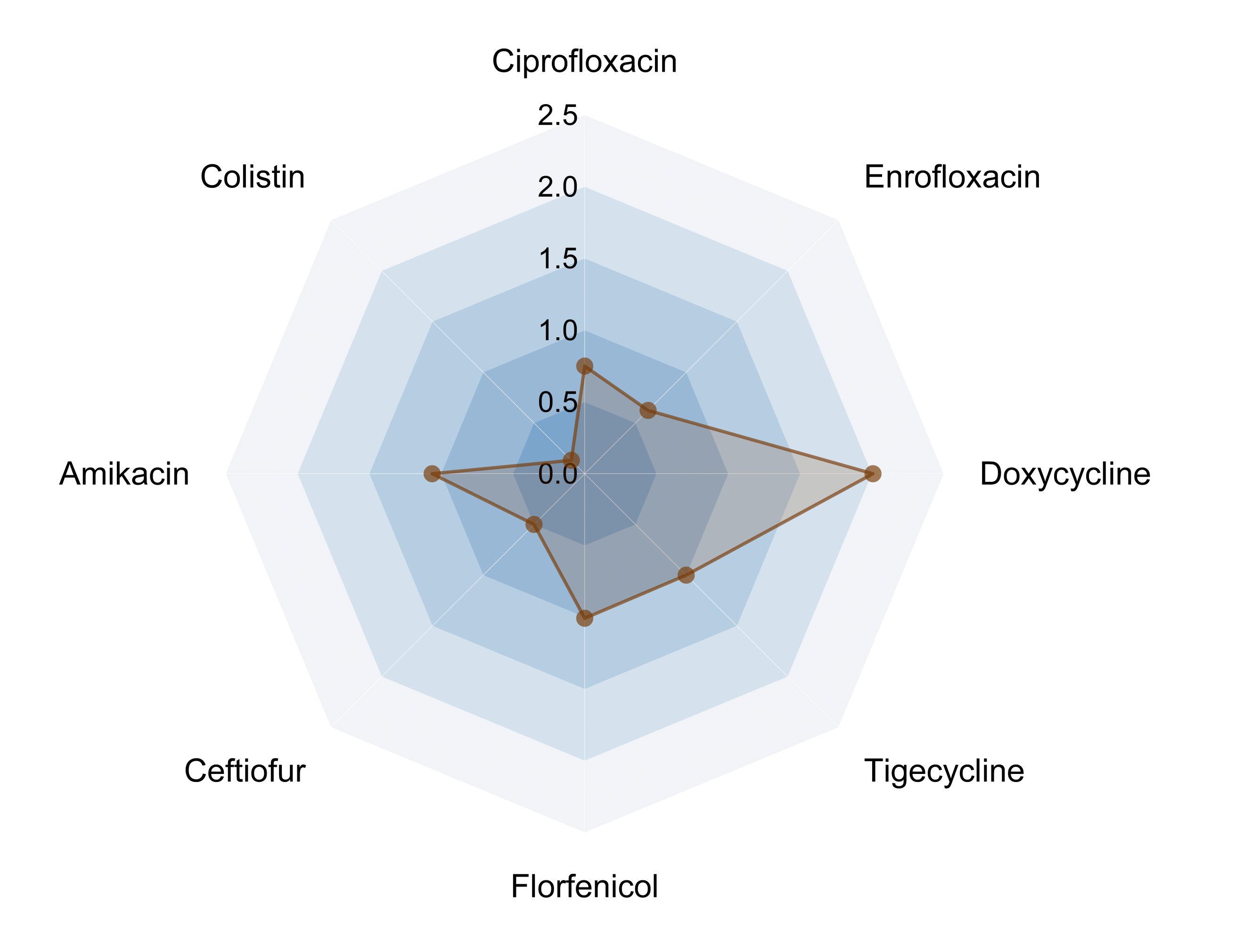


Figure S1. Evaluation of the effect of eight antibiotics in combination with LOR. The numbers in the figure represent FICI.


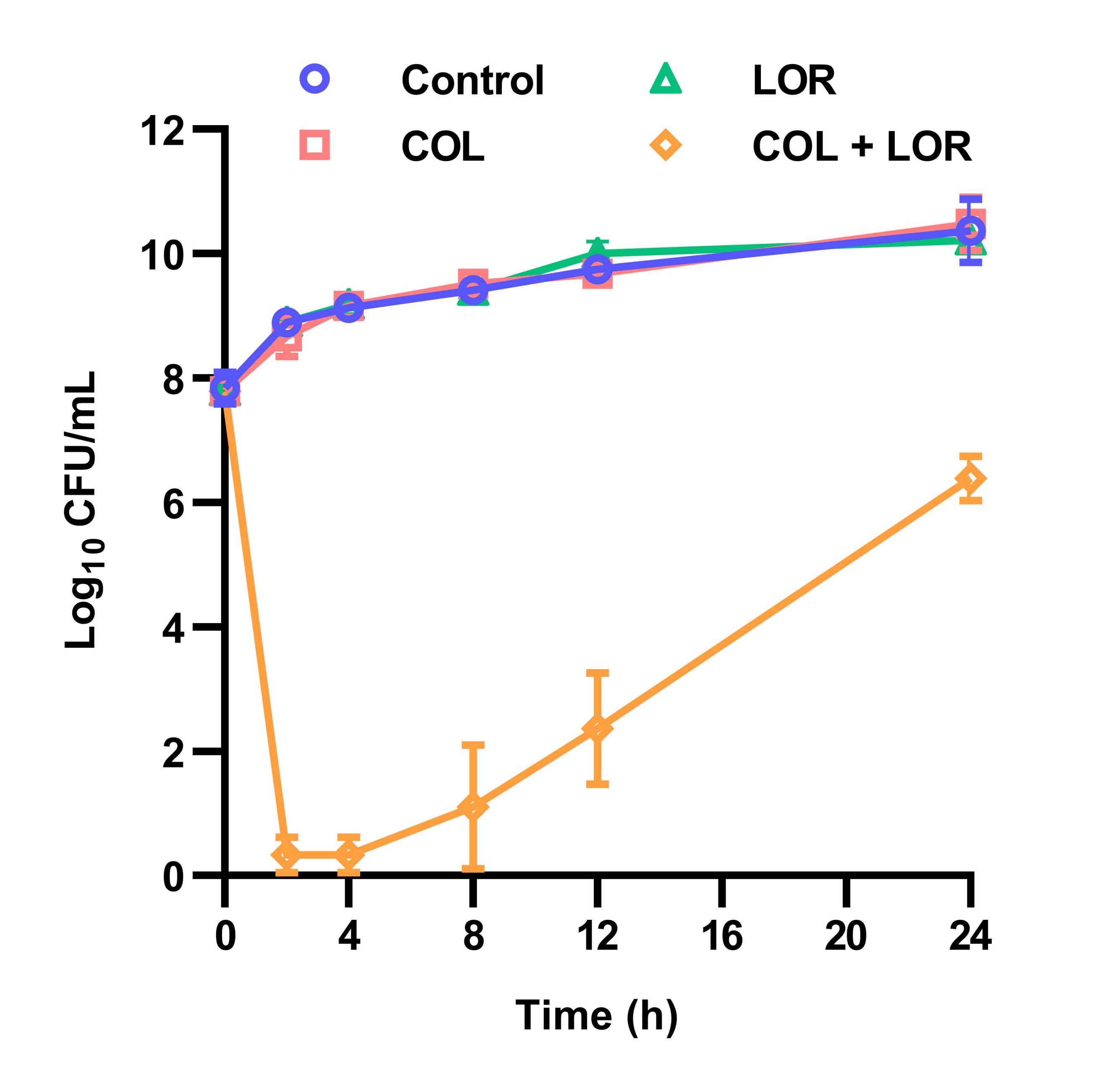


Figure S2. Time-kill curves of *K. pneumoniae* KP1. The control group was treated without drug. In the COL group, 1/2 MIC (8 μg/mL) COL was added alone. In LOR group, 1/16 MIC (32 μg/mL) LOR was added alone. In the COL + LOR group, a combination of 1/2 MIC COL and 1/32 MIC LOR was used.


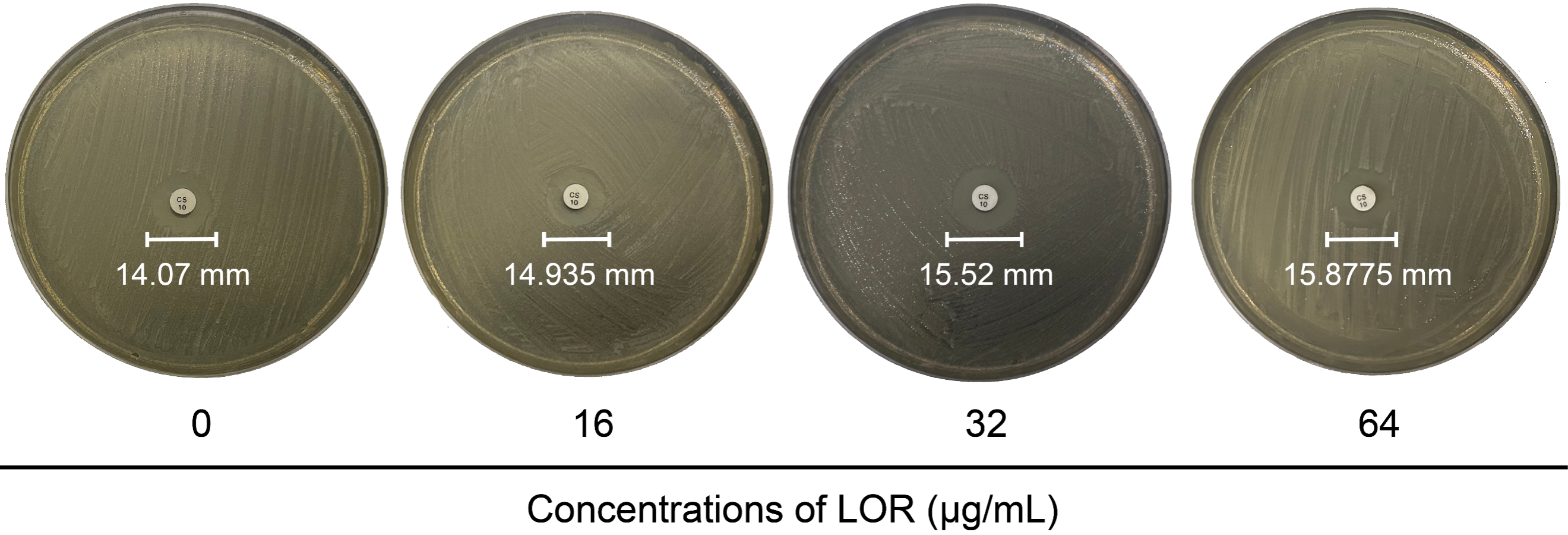


Figure S3. An intuitive diagram of the antibacterial zone test.


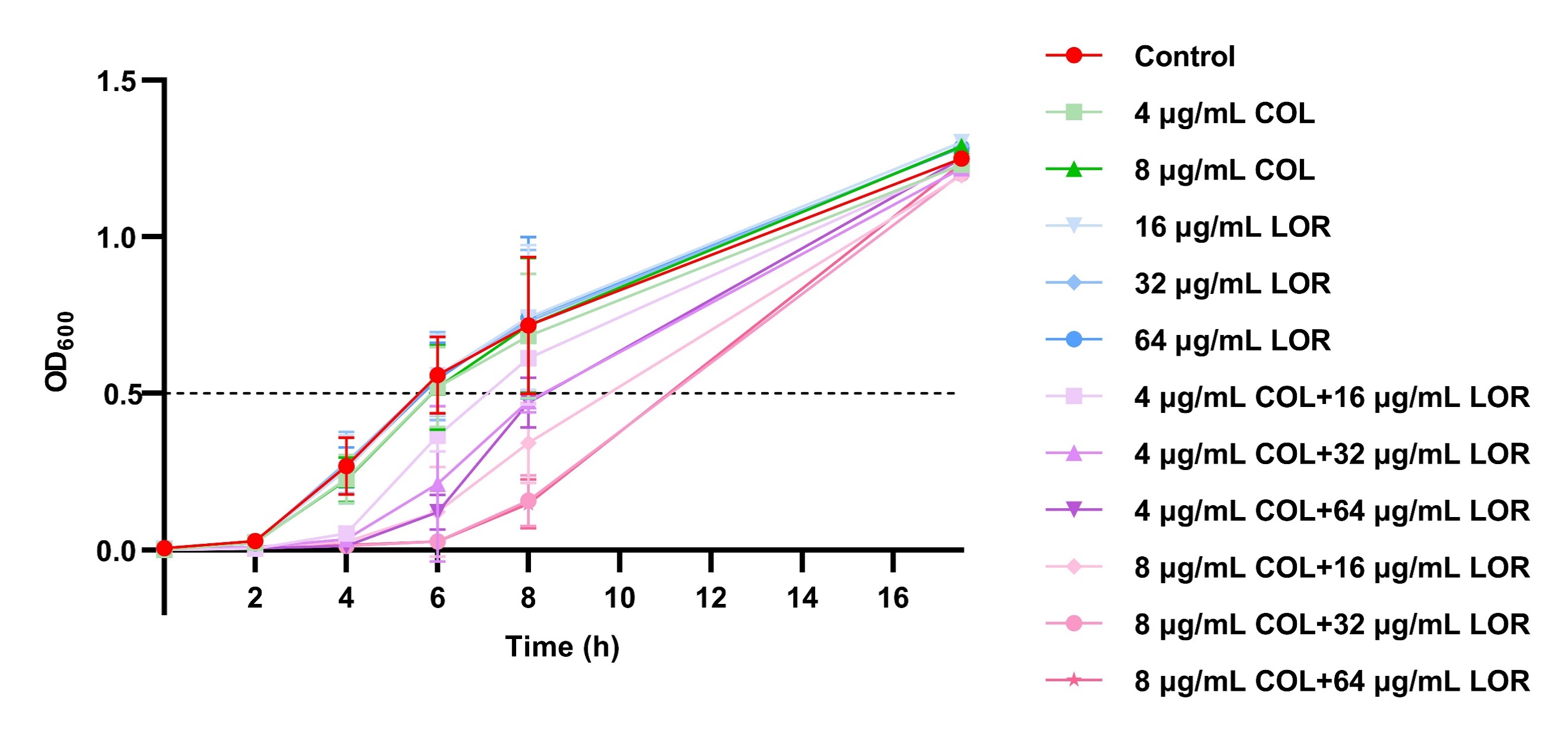


Figure S4. Bacterial regrowth after withdrawal of LOR, COL, or their combination.


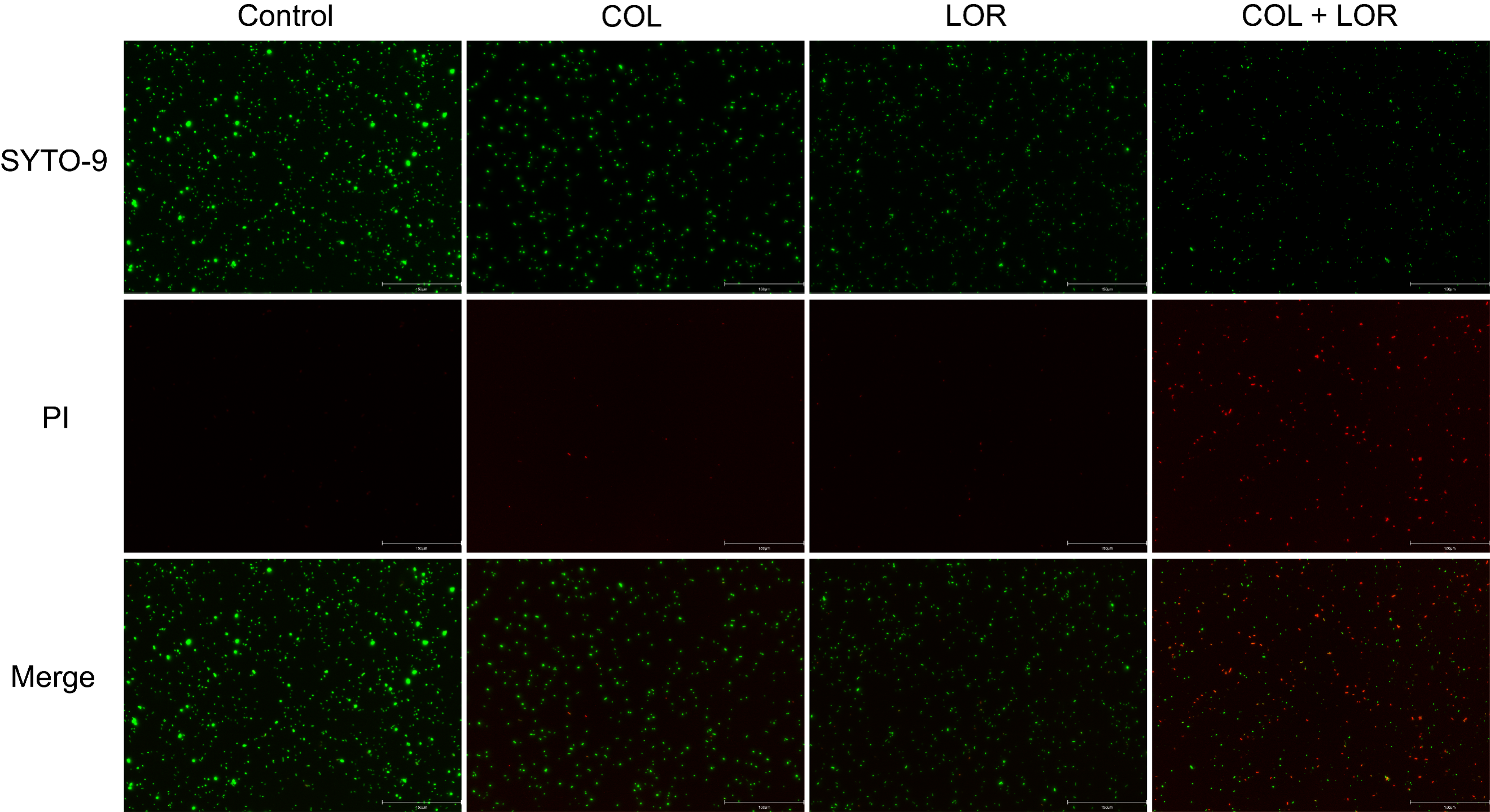


Figure S5. Fluorescence microscope images of *K. pneumoniae* JX21CTR26 under the treatment of COL (8 μg/mL), LOR (32 μg/mL) alone and its combination after live/dead staining. Live cells stain green (e.g., SYTO 9), whereas dead cells stain red (e.g., PI).


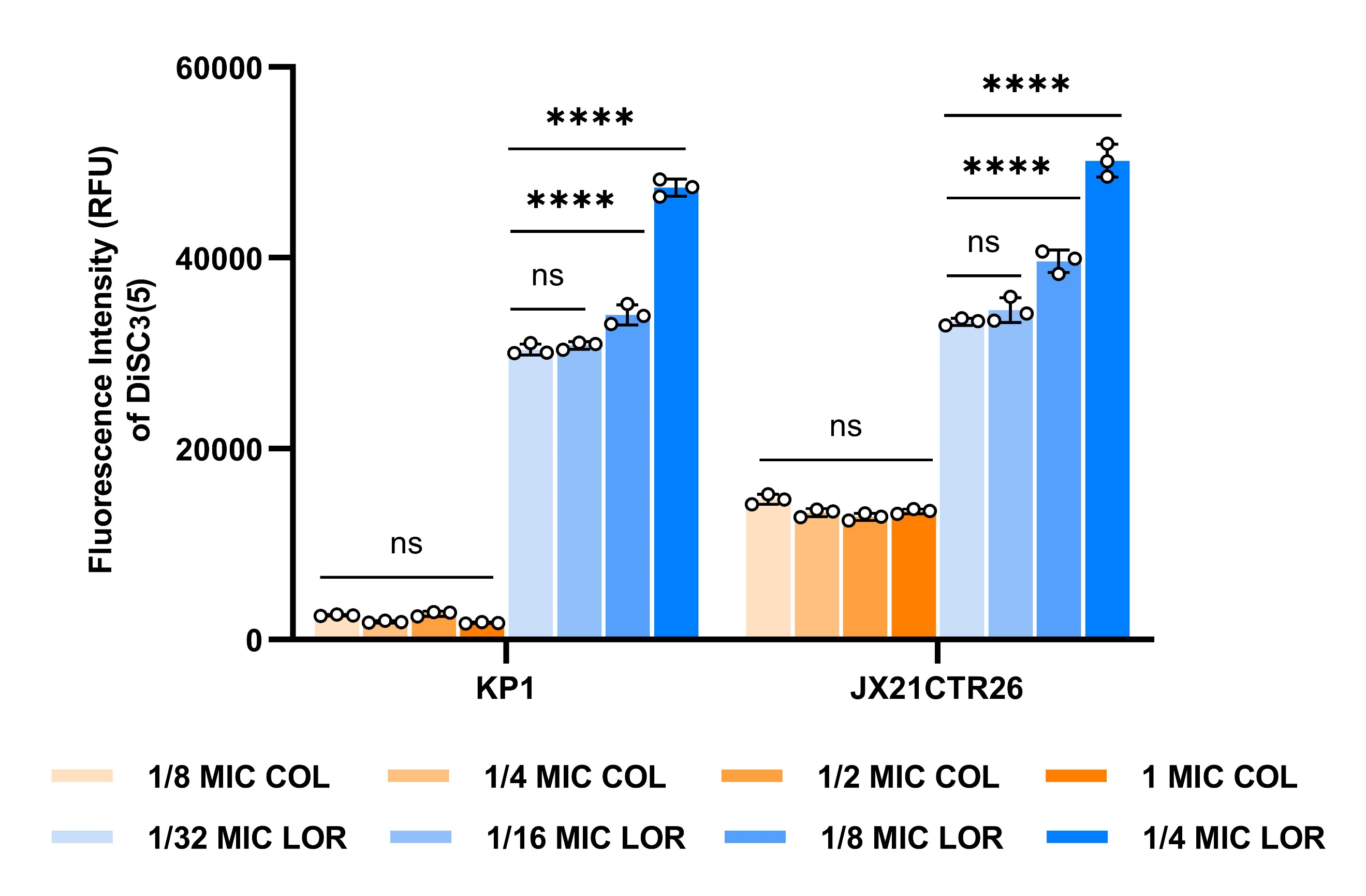


Figure S6. Separate testing of membrane potential changes in COL or LOR with changes in concentration


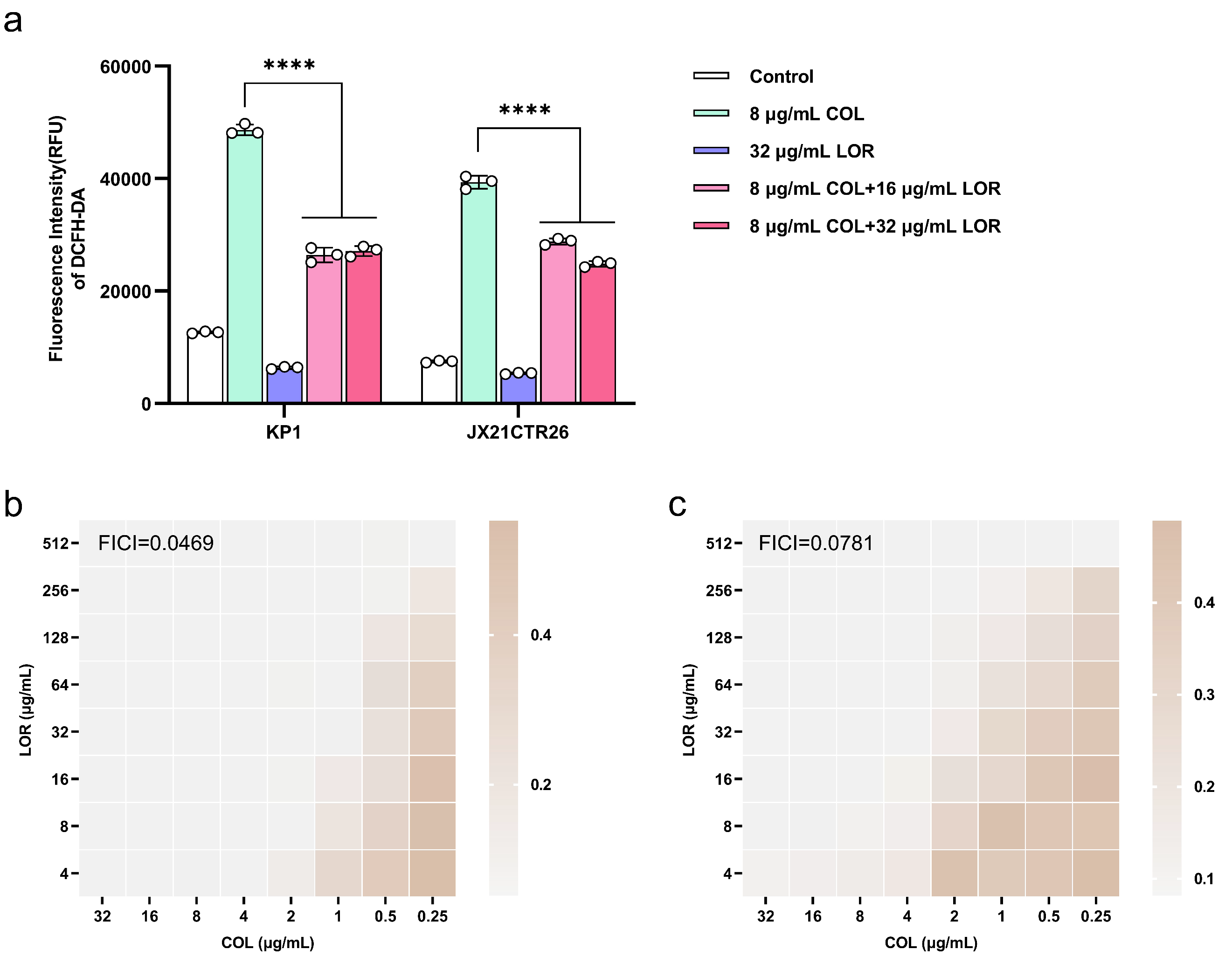


Figure S7. Evaluation of bacterial oxidative damage. (a) Changes in ROS levels after treatment with different concentrations of LOR, COL, or their combination. Susceptibility results of COL in combination with LOR against KP1 (b) and JX21CTR26 (c) after exogenous addition of ROS scavenger NAC. The dark region represents a higher cell density.
